# Physiologic upper limits of pore size of different blood capillary types and another perspective on the dual pore theory of microvascular permeability

**DOI:** 10.1186/2040-2384-2-14

**Published:** 2010-08-11

**Authors:** Hemant Sarin

**Affiliations:** 1 National Institute of Biomedical Imaging and Bioengineering, National Institutes of Health, Bethesda, Maryland 20892, USA

## Abstract

**Background:**

Much of our current understanding of microvascular permeability is based on the findings of classic experimental studies of blood capillary permeability to various-sized lipid-insoluble endogenous and non-endogenous macromolecules. According to the classic small pore theory of microvascular permeability, which was formulated on the basis of the findings of studies on the transcapillary flow rates of various-sized systemically or regionally perfused endogenous macromolecules, transcapillary exchange across the capillary wall takes place through a single population of small pores that are approximately 6 nm in diameter; whereas, according to the dual pore theory of microvascular permeability, which was formulated on the basis of the findings of studies on the accumulation of various-sized systemically or regionally perfused non-endogenous macromolecules in the locoregional tissue lymphatic drainages, transcapillary exchange across the capillary wall also takes place through a separate population of large pores, or capillary leaks, that are between 24 and 60 nm in diameter. The classification of blood capillary types on the basis of differences in the physiologic upper limits of pore size to transvascular flow highlights the differences in the transcapillary exchange routes for the transvascular transport of endogenous and non-endogenous macromolecules across the capillary walls of different blood capillary types.

**Methods:**

The findings and published data of studies on capillary wall ultrastructure and capillary microvascular permeability to lipid-insoluble endogenous and non-endogenous molecules from the 1950s to date were reviewed. In this study, the blood capillary types in different tissues and organs were classified on the basis of the physiologic upper limits of pore size to the transvascular flow of lipid-insoluble molecules. Blood capillaries were classified as non-sinusoidal or sinusoidal on the basis of capillary wall basement membrane layer continuity or lack thereof. Non-sinusoidal blood capillaries were further sub-classified as non-fenestrated or fenestrated based on the absence or presence of endothelial cells with fenestrations. The sinusoidal blood capillaries of the liver, myeloid (red) bone marrow, and spleen were sub-classified as reticuloendothelial or non-reticuloendothelial based on the phago-endocytic capacity of the endothelial cells.

**Results:**

The physiologic upper limit of pore size for transvascular flow across capillary walls of non-sinusoidal non-fenestrated blood capillaries is less than 1 nm for those with interendothelial cell clefts lined with zona occludens junctions (i.e. brain and spinal cord), and approximately 5 nm for those with clefts lined with macula occludens junctions (i.e. skeletal muscle). The physiologic upper limit of pore size for transvascular flow across the capillary walls of non-sinusoidal fenestrated blood capillaries with diaphragmed fenestrae ranges between 6 and 12 nm (i.e. exocrine and endocrine glands); whereas, the physiologic upper limit of pore size for transvascular flow across the capillary walls of non-sinusoidal fenestrated capillaries with open 'non-diaphragmed' fenestrae is approximately 15 nm (kidney glomerulus). In the case of the sinusoidal reticuloendothelial blood capillaries of myeloid bone marrow, the transvascular transport of non-endogenous macromolecules larger than 5 nm into the bone marrow interstitial space takes place via reticuloendothelial cell-mediated phago-endocytosis and transvascular release, which is the case for systemic bone marrow imaging agents as large as 60 nm in diameter.

**Conclusions:**

The physiologic upper limit of pore size in the capillary walls of most non-sinusoidal blood capillaries to the transcapillary passage of lipid-insoluble endogenous and non-endogenous macromolecules ranges between 5 and 12 nm. Therefore, macromolecules larger than the physiologic upper limits of pore size in the non-sinusoidal blood capillary types generally do not accumulate within the respective tissue interstitial spaces and their lymphatic drainages. In the case of reticuloendothelial sinusoidal blood capillaries of myeloid bone marrow, however, non-endogenous macromolecules as large as 60 nm in diameter can distribute into the bone marrow interstitial space via the phago-endocytic route, and then subsequently accumulate in the locoregional lymphatic drainages of tissues following absorption into the lymphatic drainage of periosteal fibrous tissues, which is the lymphatic drainage of myeloid bone marrow. When the ultrastructural basis for transcapillary exchange across the capillary walls of different capillary types is viewed in this light, it becomes evident that the physiologic evidence for the existence of aqueous large pores ranging between 24 and 60 nm in diameter in the capillary walls of blood capillaries, is circumstantial, at best.

## Introduction

The transvascular exchange of blood plasma water and lipid-insoluble small molecules such as electrolytes and non-electrolytes, and in some cases, of endogenous macromolecules such as peptides (i.e. hormones) and small globular proteins (i.e. albumin), between the tissue blood capillary microvasculature and tissue interstitium, takes place across water-filled channels, or aqueous pores, in the capillary wall. Since several different-sized of aqueous pores exist in the endothelial cell lining layer of the capillary wall of all blood capillary types, these different pore populations constitute the potential parallel transport pathways for the transcapillary exchange of endogenous substances across the capillary walls of all blood capillaries. One of these is the aqua(glycerol)porin aqueous small pore population within the cell membranes of endothelial cells that line the capillary walls of several different capillary types[1-4]. However, since aqua(glycerol)porins are relatively narrow channels, being less 0.5 nm wide, only limited transcapillary flow of water and lipid-insoluble small molecules can take place through this sub-population of aqueous small pores. Therefore, in all blood capillary types, the greatest proportion of water and endogenous lipid-insoluble molecule transcapillary exchange takes place across other larger, less restrictive, aqueous small pores in the capillary wall, such as: (1) the pores in the interendothelial cell junctions of non-sinusoidal non-fenestrated blood capillaries with macula occludens interendothelial cell junctions, which permit the transcapillary passage of endogenous lipid-insoluble molecules up to 5 nm in diameter; and (2) the pores in the fenestrated endothelial cell membranes (diaphragmed fenestrae) of non-sinusoidal fenestrated blood capillaries with diaphragmed fenestrae, which permit the transcapillary passage of endogenous lipid-insoluble molecules between 6 and 12 nm in diameter.

Over the years, tissue blood capillary permeability to water and lipid-insoluble molecules has been studied by several different methodologies. The investigations that have yielded the most information on the ultrastructural and physiologic basis for tissue blood capillary microvascular permeability include: (1) the transcapillary flow rates of perfused endogenous molecules of various sizes across the capillary walls of isolated cat hind-limb capillary microvasculature with the isogravometric osmotic transient method[5-7], and single intestinal mesentery capillaries with the micro-injection micro-occlusion technique[8-10]; and (2) the transcapillary accumulation of systemically administered non-endogenous molecules of various sizes, including labeled denatured proteins (radio-iodinated albumin and immunoglobulin), dextrans, and plastic nanoparticles, in the lymphatic drainage of various body regions[11-13]. Although the hydraulic permeability coefficient (*L*_p_) of the capillary wall varies over a wide range across tissue blood capillary beds, the capillary wall osmotic reflection coefficient (σ) to albumin (diameter ~7 nm[14]), which is the fraction of albumin reflected at the level of the capillary wall, is close to 1 for several different tissue capillary beds. These findings suggest that the differences in the microvascular permeabilities of different tissue blood capillaries are attributable primarily to differences in the total porous surface area available for transcapillary exchange, and that there is a remarkable conservation in the sizes of the pores in the capillary walls of different tissue blood capillaries. The findings of these studies on microvascular permeability, taken altogether, provide strong evidence for the existence of a single population of aqueous 'small' pores in the capillary walls of most tissue blood capillaries that restrict the transvascular flow of endogenous macromolecules larger in size than albumin. However, the findings of the lymph flow studies provide only circumstantial evidence for the existence of an additional population of aqueous 'large' pores, or 'capillary leaks', in the capillary walls of all tissue blood capillaries that range between 24 and 60 nm in diameter[12,13]. Furthermore, as of yet, there is no conclusive morphological evidence in support of the existence of the large pore population in the capillary walls of tissue blood capillaries[15], other than in the case of the capillary walls of hepatic tissue blood capillaries, in which there exist aqueous large pores upwards of a 100 to 200 nm in diameter[16,17].

In the absence of conclusive morphological evidence for the existence of the aqueous large pore population, the 'vesiculo-vacuolar organelles' found in the cytoplasm of the endothelial cells of most tissue blood capillary types have been assigned this role[18,19], although these organelles do not form bona fide transendothelial channels through endothelial cells. Furthermore, since the endothelial cells of most blood capillaries do not actively phago-endocytose macromolecules at high rates[20], it is expected that there would be very limited transcapillary phago-endocytic transport and transvascular release, or 'spill-over', of macromolecules into the interstitial spaces of tissues supplied by such blood capillary types. However, it is important to note that this is not the case in myeloid (red) bone marrow and hepatic blood capillaries, since the capillary walls of these blood capillaries are lined by reticuloendothelial cells, which phago-endocytose non-endogenous molecules at high rates[20-22], particularly those non-endogenous macromolecules that are not rapidly cleared from blood circulation via phagocytosis by hepatic Kupffer macrophages and splenic red pulp macrophages[20-33]. Non-endogenous macromolecules with less immunogenic surfaces, such as dextran and polyethylene glycol coated nanoparticles, that are less than 60 nm in diameter can evade phagocytosis by hepatic Kupffer macrophages and splenic red pulp macrophages[34-38]. For this reason, such non-endogenous macromolecules remain in blood circulation for a sufficiently long time to be phago-endocytosed efficiently by the capillary wall lining reticuloendothelial cells of myeloid bone marrow blood capillaries to accumulate to high concentrations within the myeloid bone marrow interstitial spaces[34-39], which is the basis for their clinical use as bone marrow imaging agents.

Since the capillary walls of myeloid bone marrow blood capillaries lack aqueous large pores[40-46], the primary route by which non-endogenous macromolecules larger than 5 nm in diameter can distribute into the bone marrow interstitial spaces and enter the locoregional lymphatic drainages is via the phago-endocytic transfer of particles into bone marrow interstitial spaces and then the absorption of macromolecules into the lymphatic drainage of periosteal fibrous tissues[47-50], which is the lymphatic drainage of myeloid bone marrow. Therefore, the presence of intravenously administered dextrans as large as approximately 24 nm in diameter in cervical and lower extremity lymphatic drainages is attributable to the accumulation of the dextran nanoparticles, first, in the transcapillary filtrates of myeloid bone marrow interstitial spaces, and then, in the lymphatic drainages of locoregional periosteal fibrous tissues. Therefore, when non-endogenous macromolecules are used as test substances to measure capillary permeability to macromolecules in lymph flow studies of microvascular permeability[11-13,51-54], these non-endogenous test substances accumulate in the locoregional lymphatics of various tissues upon phago-endocytic transfer across myeloid bone marrow blood capillaries, and subsequent absorption into the initial lymphatics of the local periosteal fibrous tissues. Due to a paucity of specific data on myeloid bone marrow sinusoidal capillary wall surface area, and on its hydraulic and osmotic reflection coefficients, at this time, it is not possible to determine the overall contribution of the phago-endocytic transcapillary transport pathway of myeloid bone marrow blood capillaries. However, it has been possible to formulate the proposed hypothesis following critical appraisal of the currently available morphological data on sinusoidal blood capillary wall ultrastructures in context of the available physiologic data on the endogenous macromolecule and non-endogenous nanoparticle uptake and distribution in tissues and organs supplied by sinusoidal blood capillaries.

In this study, the different tissue blood capillary types were classified on the basis of differences in the physiologic upper limits of pore size for transcapillary exchange of lipid-insoluble molecules in order to highlight the differences in the transcapillary routes for the transvascular transport of endogenous and non-endogenous macromolecules across the capillary walls of non-sinusoidal and sinusoidal blood capillaries. When the ultrastructural basis for transcapillary exchange is viewed in this light, it becomes evident that there is little physiologic evidence for the existence of the aqueous large pore population in the capillary walls of blood capillaries.

## Methods

The findings and published data of studies on blood capillary wall ultrastructure and capillary microvascular permeability to lipid-insoluble endogenous and non-endogenous molecules from the 1950s to the present were reviewed. These studies included: (1) electron microscopy studies on the morphologies of blood capillary microvasculatures in different tissues; (2) tracer-based electron microscopy studies on the permeability of blood capillary microvasculature to systemically infused or perfused tracer molecules of various sizes including ionic lanthanum (diameter < 1 nm)[55-57], colloidal lanthanum (diameter ~2 nm)[55-57], horseradish peroxidise (diameter ~4.6 nm)[58], hemoglobin (diameter ~6.4 nm)[14], ferritin (diameter ~12.2 nm)[59], and dextrans[14,60]; (3) physiology studies on the permeability of isolated single capillaries and on the microvascular permeability of body regions perfused with endogenous molecules of various sizes including myoglobin (diameter ~4 nm)[7] and albumin (diameter ~7 nm)[14]; and (4) immunolocalization studies of endogenous macromolecules of various sizes including albumin and immunoglobulins[14]. When pertinent physiologic data on the upper limit of pore size for a tissue blood capillary bed was unavailable, the tissue blood capillary bed was primarily classified on the basis of the capillary wall morphology.

## Results

### General

**Figure 1:**
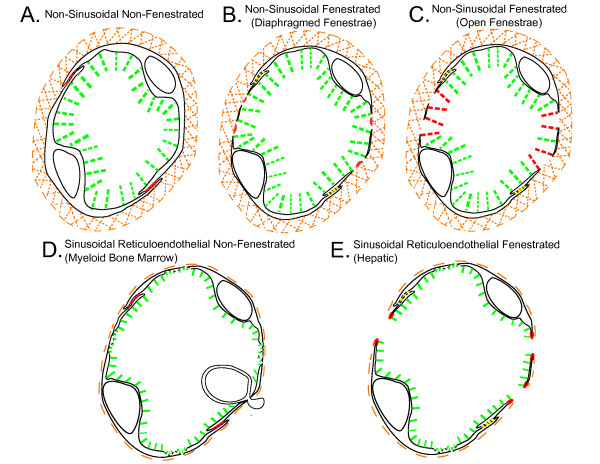
**Schematic depictions of the capillary wall ultrastructure in different blood capillary microvasculatures**. Shown in red are the anatomic sites in the capillary walls of the respective blood capillary types that are the primary pathways for transvascular flow and transport across the capillary wall, and as such, constitute the ultrastructural determinants of the physiologic upper limit of pore size to transvascular flow. The green pillars that emanate from the luminal surface of the endothelial lining represent the individual mucopolysaccharide fibers of the endothelial glycocalyx layer (EGL), and the orange hatched region that encircles the abluminal surface of the endothelial cell lining represents the collagenous basement layer (interna and externa). As depicted in the schematics, the capillary walls of the different types of non-sinusoidal blood capillaries are proficient in all three layers (panels A, B and C), which is not the case for the capillary walls of the sinusoidal blood capillaries of myeloid (red) bone marrow and the liver (panels D and E). Also depicted in panels D and E are the 'bristle-coated pits' of myeloid bone marrow and hepatic sinusoidal blood capillary the reticuloendothelial cells, which constitute the anatomic sites at which the phago-endocytosis of non-endogenous macromolecules occurs. A. Non-sinusoidal non-fenestrated blood capillaries B. Non-sinusoidal fenestrated blood capillaries with diaphragmed fenestrae C. Non-sinusoidal fenestrated blood capillaries with open 'non-diaphragmed' fenestrae D. Sinusoidal reticuloendothelial non-fenestrated blood capillaries of myeloid (red) bone marrow E. Sinusoidal reticuloendothelial fenestrated blood capillaries of the liver (Please view Additional files 1, 2, 3, 4 and 5 for individual Figure 1 panels A, B, C, D and E with detailed panel descriptions)

The different types of tissue blood capillary microvasculature are classified in Tables 1, 2, 3 and 4: Table 1 is the classification scheme for non-sinusoidal non-fenestrated blood capillary microvasculature, Table 2 is the classification scheme for non-sinusoidal fenestrated blood capillary microvasculature, Table 3 is the classification scheme for sinusoidal reticuloendothelial blood capillary microvasculature, and Table 4 is the classification scheme for sinusoidal non-reticuloendothelial blood capillary microvasculature. The capillary wall ultrastructures of different blood capillary microvasculatures are schematically depicted in Figure 1 (Panels A, B, C, D and E; Individual panels and detailed descriptions in Additional files 1, 2, 3, 4 and 5). The physiologic upper limit of pore size of the blood capillary, as defined here, is the hydrodynamic diameter of the largest lipid-insoluble molecule that is restricted from passing through the pores in the capillary wall, and as such, constitutes the size of the molecule to which the pores in the capillary wall are impermeable. The blood capillary wall is a three-layered structure in most types of tissue blood capillaries, which consists of the endothelial glycocalyx layer (EGL) on the luminal face[61-63], the basement membrane layer on the abluminal face[64-68], and the endothelial cell lining layer in between the glycocalyx and the basement membrane[69-78] (Figure 1, panels A, B, C, D and E; Additional files 1, 2, 3, 4 and 5). The physiologic upper limit of pore size is for any given capillary type is determined by the most restrictive layer of the capillary wall (Tables 1, 2, 3 and 4; Figure 1, panels A, B, C, D and E; Additional files 1, 2, 3, 4 and 5). For non-sinusoidal non-fenestrated blood capillaries, the pore size of the interendothelial cell junction openings delineates the physiologic upper limit of pore size[69,70,79-81] (Table 1; Figure 1, panel A; Additional file 1). For non-sinusoidal fenestrated blood capillaries with diaphragmed fenestrae, the pore size of the open spaces devoid of diaphragm membranous components delineates the physiologic upper limit of pore size[72-76,82] (Table 2; Figure 1 panel B; Additional file 2). For non-sinusoidal fenestrated blood capillaries with open 'non-diaphragmed' fenestrae, the fenestral openings are bounded by a high-concentration of glycocalyx matrix fibers; therefore, the pore size of the open spaces between the individual glycocalyx matrix fibers delineates the physiologic upper limit of pore size[61] (Table 2; Figure 1, panel C; Additional file 3). In case of the sinusoidal blood capillaries of the liver that have open fenestrae, the boundaries of the fenestral openings lack an appreciable concentration of glycocalyx matrix fibers, and the capillary wall lacks a basement membrane; therefore, the pore size of the fenestral openings delineates the physiologic upper limit of pore size[16,17,83] (Table 3; Figure 1, panel E; Additional file 5).

**Table 1:** Classification of non-sinusoidal non-fenestrated blood capillary microvasculature

NON-SINUSOIDAL CAPILLARY TYPE	Primary anatomic sites of transvascular flow	Determinants of physiologic pore size	Physiologic upper limit of pore size	Representative tissue microvascular beds
**NON-FENESTRATED**	•Non-fenestrated endothelial cells •Continuous anionic basement membrane rich in sulphated proteoglycans[64,86] •Anionic glycocalyx matrix layer on endothelial cell surfaces & interendothelial cell clefts rich in sialyated glycoproteins[69,142,152-154]			

**Non-fenestrated blood capillary with tight junctions**	**Zona occludens interendothelial junctions** •Tight opposition of adjacent endothelial cell membranes at junctions [70,92,93,155]	•Zona Occludens interendothelial cell junctions in series constitute an absolute barrier to the transvascular flow of macromolecules	**< 1 nm**	•Retinal [156,157] •Brain-Spinal Cord [85,93,95,96,158,159] •Nerve Endoneurium [160] •Enteric Nervous System [91] •Lymphoid tissue Cortex [161-163]

**Non-fenestrated blood capillary with loose junctions**	**Macula occludens interendothelial junctions** •Loose opposition of adjacent endothelial cell membranes at junctions [79,80]	•Macula Occludens interendothelial cell junctions in series constitute a relative barrier to the transvascular flow of macromolecules	**~5 nm**	•Skin* [164,165] •Muscle [7,79,80,90] •Cortical Bone [88] •Adipose tissue [87] •Lung [81] •Intestinal Mesentary [15,71] •Develop. Ovarian Follicle [94]

**Table 2:** Classification of non-sinusoidal fenestrated blood capillary microvasculature

NON-SINUSOIDAL CAPILLARY TYPE	Primary anatomic sites of transvascular flow	Determinants of physiologic pore size	Physiologic upper limit of pore size	Representative tissue microvascular beds
**FENESTRATED**	•Fenestrated endothelial cells •Continuous anionic basement membrane rich in sulphated proteoglycans[64,86] •Anionic glycocalyx matrix of endothelial cell surfaces & clefts rich in sialyated glycoproteins & of fenestrated spaces rich in sulphated proteoglycans[166-170]			

**Fenestrated blood capillary with diaphragmed fenestrae**	**Diaphragmed fenestrae** •Diameters of fenestrae range between 60 and 80 nm •Widths of closed membranous central diaphragms range between 10 and 30 nm •Eight to twelve, 2 to 7 nm wide, outwardly radiating membranous septae from central diaphragm •Arc widths of fenestrated open spaces between ~6 and ~12 nm [72-76,82]	•Arc widths of open spaces devoid of membranous components (central diaphragm and septae) delineate the upper limits of pore size •Diaphragms of diaphragmed fenestrae constitute the barriers to the transvascular flow of macromolecules •Anionic glycocalyx matrix over fenestrated spaces charge barrier to the transvascular flow of anionic macromolecules	**Between 6 & 12 nm**	•Skin* [164,165] •Testis [171-173] •Connective tissue [174,175] •Eye Choriocapillaris [82,102-104,176,177] •Exocrine Glands [105-108] •Kidney Peritubular [72,178] •Endocrine Glands [73,106,179-185] •Intestinal Mucosa [186-191] •Peripheral Ganglia [158,192-194] •Nerve Epineurium [160] •Circumventricular Organs [109,110,195-199] •Choroid Plexus [109-113] •Pre-Ovulatory Follicle [114,115] •Eye Ciliary Process [116-120]

**Fenestrated blood capillary with open fenestrae**	**Open fenestrae** •Open 'non-diaphragmed' fenestrae with avg. diameters of 65 nm devoid of the central diaphragm & septae [61,72,200]	•Narrow interspacing of glycocalyx matrix fibers is the barrier to the transcapillary flow of macromolecules larger than ~15 nm in diameter	**~15 nm**	•Kidney Glomerulus [60,72,101,123-126,201,202]╬ ╬Slit diaphragms at the level of podocyte foot processes restrict the filtration of plasma proteins larger than 6 nm in diameter (i.e. hemoglobin, albumin)

**Table 3:** Classification of sinusoidal reticuloendothelial blood capillary microvasculature

SINUSOIDAL CAPILLARY TYPE	Primary anatomic sites of transvascular flow	Ultrastructural determinants of transvascular transport	Physiologic upper limit of pore size
**RETICULO-ENDOTHELIAL**	•Endothelial cells w/high levels of phago-endocytosis[130,131] •Basement membrane commonly absent[64,77,203] •Patchy anionic glycocalyx of sialyated glycoproteins at non-endocytic sites[132-134] deficient in hyaluronan[137-140]		

**Hepatic sinusoidal blood capillary**	**Open fenestrae** •Diameters of open fenestrae variable across mammalian species: 1)human & rabbit: avg. diameter ~105 nm (range 50 to 180 nm) [17,83] 2) mouse & rat: avg. diameter ~135 nm (range 50 to 280 nm) [16,83] •Absence of basement membrane underlying fenestrae and lack of glycocalyx matrix fibers within fenestrae [204] •Phago-endocytic phenotype of endothelial cells [205-207]	•Absence of basement membrane underlying fenestral openings and relative lack of glycocalyx matrix fibers in the vicinity of the fenestral openings renders fenestrae permeable to macromolecules as large as the diameters of the fenestrae themselves [16,17] •Open fenestrae constitute the transvascular pathway for the passage of macromolecules into the interstitial hepatic Space of Disse •Phago-endocytosis and release of non-endogenous macromolecules into the hepatic interstitium [141] •Wide size range of nanoparticles [136,208-210]*	***Transvascular flow*** **~180 nm *(Human, Rabbit)*** **~280 nm ***(Mouse, Rat)* ***Phago-endocytic ***(Non-endogenous) Wide Range*

**Myeloid bone marrow sinusoidal blood capillary**	**Interendothelial junctions** •Endothelial cells non-fenestrated except during transcellular passage of blood cells across endothelial cell membrane when cells transiently fenestrated [40-42] •Macula occludens interendothelial junctions [42-44] •Phago-endocytic phenotype of endothelial cells [45,46,211]	•Transvascular flow of macromolecules smaller than ~5 nm into the bone marrow interstitial space across maculae occludens interendothelial junctions • Phago-endocytosis and release of non-endogenous macromolecules into bone marrow interstitium [34,35] •Narrow size range of nanoparticles [36-38]**	***Transvascular flow*** **~5 nm** ***Phago-endocytic ***(Non-endogenous) Narrow Range**

**Table 4:** Classification of sinusoidal non-reticuloendothelial blood capillary microvasculature

SINUSOIDAL CAPILLARY TYPE	Primary anatomic sites of transvascular flow	Ultrastructural determinants of transvascular transport	Physiologic upper limit of pore size
**NON-RETICULO-ENDOTHELIAL**	•Endothelial cells w/low levels of phago-endocytosis[20-22] •Basement membrane discontinuous[23-25] •Thin anionic glycocalyx over endothelial cell surfaces of both capillary types[26]		

**Splenic red pulp arterial blood capillary (Terminal)**	**Terminal capillary ending** •Terminal capillary ending openings ~5 microns (μm) in diameter [27,28] •Basement membrane sparse and intermittent •Macrophages in the terminal arterial pericapillary sheath and within the splenic red pulp reticular meshwork [29,30,212]	•Terminal arterial capillary network of the splenic red pulp reticulum constitutes the primary mode of splenic filtration •Macromolecules as large as 5 μm pass into splenic red pulp reticulum through terminal capillary ending openings •Exogenous macromolecules phagocytosed by macrophages in the terminal arterial pericapillary sheath and in the red pulp reticulum	**~5 μm**

**Splenic red pulp venous blood capillary (Sinus)**	**Interendothelial slits** •Cuboidal endothelial cells •Interendothelial slits between apical and basal adherens junctions •Basement membrane ringed and belts of basement membrane rings 2-3 μm apart [25] •Slits closed except during active blood cell migration [25,31] and macrophage phagocytosis [32]	•Few direct connections exist between splenic arterioles and venous capillaries and constitutes the minor pathway in splenic filtration •Exogenous macromolecules in sinus lumen phagocytosed at level of the interendothelial slits by finger-like pseudopodia of splenic pulp reticulum macrophages	**__**

### Non-sinusoidal versus sinusoidal blood capillary microvasculature

Blood capillaries can be classified as non-sinusoidal or sinusoidal based on differences in the ultrastructure of the basement membrane layer of the capillary wall. The main distinction between non-sinusoidal and sinusoidal capillaries is the presence or absence of a continuous basement membrane layer (Figure 1, panels A, B and C; Additional files 1, 2 and 3 versus Figure 1, panels D and E; Additional files 4 and 5). Non-sinusoidal blood capillaries have traditionally been termed 'continuous' capillaries due to the presence of a continuous basement membrane layer[84]. These blood capillaries are classified as 'non-sinusoidal' capillaries here is to minimize ambiguity, since these capillaries are further sub-classified as either non-fenestrated or fenestrated based on the absence or presence of fenestrations within the endothelial cells of the endothelial cell lining layer. The basement membrane layer of both types of non-sinusoidal capillaries is typically between 60 and 100 nm in thickness, and is composed of collagen type IV proteins and glycoproteins interlinked by proteoglycans with heparan sulphate glycosaminoglycan side chains, which are concentrated at the anionic sites of the basement membrane[64,67,68].

Although the blood capillaries of most tissues are non-sinusoidal, the blood capillaries of certain tissues, the myeloid bone marrow, liver, and spleen, are sinusoidal, as these capillary types are deficient in the basement layer[16,17,24,25,27,29-31,41,42]. In the case of myeloid bone marrow and hepatic sinusoidal capillaries, or sinusoids, the basement membrane layer is generally absent, or sparsely distributed along the capillary path length, therefore, discontinuous[16,17,41,42,44] (Figure 1, panels D and E; Additional files 4 and 5). In the case of splenic sinusoidal capillaries, the basement membrane of both splenic red pulp terminal arterial capillary endings and splenic red pulp venous capillaries (sinuses) is discontinuous, but for different reasons. In the case of splenic red pulp terminal arterial capillary endings, the basement membrane is sparsely distributed around the capillary ending[24,27,29,30]; whereas, in the case of splenic red pulp venous capillaries (sinuses), the basement membrane of is comprised of ringed belts of basement membrane with individual belts 2 to 3 μm apart[23,25,31,32]. The discontinuities in the basement membrane layer of myeloid bone marrow sinusoids and splenic venous sinuses render these sinusoidal capillaries less restrictive to blood cell transmigration; as such, the less restrictive capillary wall phenotype of these sinusoidal capillaries is consistent with the functional roles that these tissues play in hematopoiesis and in the immune response of mononuclear phagocytic system, which requires that monocytes and phagocytes residing within the respective interstitial spaces are efficiently mobilized when necessary.

### Non-sinusoidal non-fenestrated blood capillaries: Ultrastructure and the physiologic upper limit of pore size

The endothelial cell lining layer of non-sinusoidal non-fenestrated blood capillaries is continuous, as is the basement membrane layer[70,80,81,85,86]. Since there are no fenestrations within the endothelial cells of non-sinusoidal non-fenestrated capillaries, the interendothelial cell clefts constitute the primary pathways for transvascular flow across the capillary wall[15,71,79-81,85,87-94] (Table 1; Figure 1, panel A; Additional file 1). Although the widths of the interendothelial cell clefts at the level of the endothelial cell surface are approximately 20 nm, the diameters of pore openings in the interendothelial cell junctions of the cleft are much narrower. In the case of zona occludens tight junctions, the endothelial cell membranes are tightly opposed at sites of most junctions, as there are no gaps of measurable dimensions at the sites of these junctions with electron microscopy[70,85,92,93]. Although there can be occasional breaks in the junctional strand of one or more zona occludens junctions within any given interendothelial cell cleft, this is almost never the case for the entire series of junctions within the cleft; therefore, a series of zona occludens junctions in a cleft constitute an absolute barrier to the transvascular passage of macromolecules[70]. Therefore, in the physiologic state *in vivo*, the capillary walls of non-fenestrated capillaries with zona occludens tight junctions only permit the transvascular flow of small molecules, and completely restrict the transvascular flow of macromolecules; as such, the physiologic upper limit of pore size of these capillaries is less than 1 nm[85,92,93,95,96]. Whereas zona occludens tight junctions are only permeable to small lipid-insoluble molecules, macula occludens loose junctions are open junctions with pore diameters between 4 and 5 nm on electron micrographs[79,80], and are permeable to macromolecules as large as myoglobin (diameter ~4 nm) and horseradish peroxidise (diameter ~4.6 nm)[79-81,90,94]. Although there can occasionally be significant breaks in the junctional strands of one or more macula occludens junctions within the interendothelial cell cleft[71], this is also almost never the case for the entire series of macula occludens junctions in the cleft. Therefore, the physiologic upper limit of pore size in the capillary walls of non-fenestrated capillaries with macula occludens loose junctions is approximately 5 nm.

### Non-sinusoidal fenestrated blood capillaries: Ultrastructure and the physiologic upper limit of pore size

The basement membrane layer of non-sinusoidal fenestrated blood capillaries is continuous (Table 2; Figure 1, panels B and C; Additional files 2 and 3), and therefore, similar to that of non-sinusoidal non-fenestrated blood capillaries[86] (Table 1; Figure 1, panel A; Additional file 1). However, the endothelial cells of the endothelial lining of non-sinusoidal fenestrated blood capillaries are fenestrated, either by diaphragmed fenestrae, or by open 'non-diaphragmed' fenestrae, with the diameters of both types of fenestrae being within the 70 nm range[72-76,82] (Table 2; Figure 1, panels B and C; Additional files 2 and 3). The induction of diaphragmed fenestrae within endothelial cells is known to be mediated primarily by vascular endothelial growth factor (VEGF), and results in the formation of fenestrae with membranous components[97-100]; whereas, the specific molecular pathways that result in the formation of the open 'non-diaphragmed' fenestrae in the endothelial cells of the non-sinusoidal fenestrated blood capillaries of the kidney glomerulus have not yet been deciphered[101]. In the case of diaphragmed fenestrae, the diaphragmed portion consists of a membranous central diaphragm between 10 and 30 nm wide, and the eight to twelve, 2 to 7 nm wide, membranous septae that radiate outward from the central diaphragm to the fenestral rim[72,74-76,82]. The arc widths of the open spaces devoid of membranous components have been measured to range between 6 and 12 nm[72,82]. The physiologic upper limit of pore size in the capillary walls of non-sinusoidal blood capillaries with diaphragmed fenestrae ranges between 6 and 12 nm, with the upper limit of pore size of eye choriocapillaris, exocrine glands and kidney peritubules being closer to 6 nm[102-108], and that of choroid plexus, pre-ovulatory follicle and eye ciliary process being closer to 12 nm[109-120]. Even though in this particular study, only the blood capillary types found in different normal healthy tissues have been formally classified, it deserves mention, that the VEGF-derived pathologic blood capillaries of solid cancers are also non-sinusoidal fenestrated blood capillaries with diaphragmed fenestrae, and that the physiologic upper limit of pore size in these blood capillaries has recently been defined as being approximately 12 nm[121,122]. The fact that the anatomically-measured arc widths of the open spaces within the diaphragmed fenestrae of the non-sinusoidal fenestrated blood capillaries from different tissues is similar to physiologically-measured sizes of the pores within these capillaries from different tissues strongly suggests that the primary physical barriers to the transvascular flow of macromolecules are the diaphragmed membranous components of the diaphragmed fenestrae. The highly anionic fibers of the endothelial glycocalyx that are rich in heparin and heparan sulphate proteoglycans are located in the vicinity of the pores of the diaphragmed fenestrae; and therefore, they constitute the primary electrostatic barriers to the transvascular flow of macromolecules with low isoelectric points.

Whereas non-sinusoidal fenestrated blood capillaries with diaphragmed fenestrae are present in a wide-variety of tissue types, non-sinusoidal fenestrated blood capillaries with open fenestrae are only known to be present in the kidney glomerulus. Even though the anatomic diameters of open fenestrae are on average approximately 65 nm, the physiologic upper limit of pore size of kidney glomeruli capillaries is approximately 15 nm[101,123-127]. This difference between the anatomic diameters of the open fenestrae and the observed physiologic upper limit of pore size is attributable to presence of narrowly interspaced endothelial glycocalyx matrix fibers flanking over the open fenestrae (Table 2; Figure 1, panel C; Additional file 3). Since the interspacing of glycocalyx matrix fibers is a maximum of 20 nm in all directions, the physiologic upper limit of pore size of open fenestrae would be a maximum of 20 nm, which depends, of course, on the thickness of the individual fibers[61,128].

The physiologic upper limit of pore size of approximately 15 nm for kidney glomeruli capillaries stated here is based on the evidence that both endogenous and non-endogenous macromolecules with hydrodynamic diameters of approximately 12 nm (native ferritin, immunoglobulin G) traverse the open fenestrae[101,123-125], while dextrans with diameters of approximately 15 nm are restricted at the level of the open fenestrae[60]. It is noted that dextrans are flexible polymers, and may not be the optimal macromolecules for the purposes of defining the upper limit of pore size; therefore, the physiologic upper limit of pore size of approximately 15 nm reported here should be considered a relative upper limit, as the actual upper limit may be closer to 13 or 14 nm. The physiologic upper limit of pore size of the kidney glomerulus capillaries is not the physiologic upper limit of pore size for renal filtration, as the filtration slit diaphragms distal to the glomerulus capillary basement, at the level of podocyte foot processes, restrict the filtration of plasma proteins larger than 6 nm in diameter, for example, hemoglobin and albumin[125,126,129].

### Sinusoidal reticuloendothelial blood capillaries of liver and myeloid bone marrow: Ultrastructure and the physiologic upper limit of pore size

The blood capillaries of the myeloid bone marrow and liver are classified as reticuloendothelial blood capillaries, since the endothelial cells of these capillaries are phago-endocytic by phenotype, as they possess a complete set of hydrolytic enzymes[130,131] (Table 3; Figure 1, panels D and E; Additional files 4 and 5). The endothelial glycocalyx layer of sialyated glycoproteins is patchy and deficient in the bristle-coated pits, which are the phago-endocytic sites[132-136]. In addition, the endothelial cells of both hepatic and myeloid bone marrow sinusoidal blood capillaries express several different uptake receptors for hyaluronan (hyaluronic acid), a high-molecular weight proteoglycan glycosaminoglycan side chain, the presence of which is necessary for maintaining the stability and integrity of the endothelial glycocalyx layer[137-140]. The fact that hyaluronan is actively removed is the most likely reason for the low concentration and patchy distribution of the anionic endothelial glycocalyx layer over the reticuloendothelial cell lining of hepatic and myeloid bone marrow sinusoidal blood capillaries.

The main distinguishing morphologic feature of hepatic and myeloid bone marrow sinusoids is the ultrastructure of the endothelial cell lining layer. The endothelial cells of hepatic sinusoids are fenestrated by open fenestrae[16,17,83] (Table 3; Figure 1, panel E; Additional file 5); whereas, the endothelial cells of myeloid bone marrow are generally non-fenestrated[41-44] (Table 3; Figure 1, panel D; Additional file 4). Therefore, in the case of hepatic sinusoids, the open fenestrae constitute the primary pathway for transvascular flow of lipid-insoluble endogenous macromolecules across the capillary wall; whereas, in the case of myeloid bone marrow sinusoids, the interendothelial cell clefts with macula occludens loose junctions constitute the primary pathway for transvascular flow of such molecules across the capillary wall. The ultrastructure of the capillary wall of hepatic sinusoidal blood capillaries differs in two important respects from the capillary wall of the non-sinusoidal blood capillaries of the kidney glomerulus, which are also lined by endothelial cells with open fenestrae. The open fenestrae in the reticuloendothelial cells of hepatic sinusoidal blood capillaries lack an appreciable concentration of glycocalyx matrix fibers in the vicinity of the fenestral openings, which renders these open fenestrae less restrictive to the transvascular flow of larger macromolecules. Furthermore, due to the absence of the underlying abluminal basement membrane layer, the open fenestrae of the hepatic sinusoidal blood capillaries permit the unrestricted passage of macromolecules as large as the widths of the fenestral openings themselves, which are the openings across which large chylomicrons and lipoproteins flow through to enter the hepatic interstitial Space of Disse. The diameters of the fenestrae in the capillary walls of the hepatic sinusoidal capillaries vary across mammalian species: The diameters of the fenestrae in the capillary walls of human hepatic sinusoidal capillaries are on average approximately 105 nm and range between 50 and 180 nm; whereas, those in the capillary walls of rodent hepatic sinusoids are on average approximately 135 nm and range between 50 and 280 nm. Therefore, the physiologic upper limit of pore size in the capillary wall of human hepatic sinusoids is approximately 180 nm, and in rodent hepatic sinusoids is approximately 280 nm[16,17]. Chylomicrons and other endogenous macromolecules can pass into the hepatic interstitium via transvascular flow across the fenestral openings, since these macromolecules are not phagocytosed by the lining reticuloendothelial cells and Kupffer macrophages of the hepatic sinusoids; however, in the case of non-endogenous macromolecules, the proportion of macromolecules that flow into the hepatic interstitium by transvascular flow across the fenestrae, and rapidly accumulate in the hepatic lymphatic drainage, constitutes the proportion not phagocytosed at the level of the capillary wall by Kupffer macrophages and the reticuloendothelial cells, which is dependent on particle dose[11,141].

The endothelial cell lining layer in the case of myeloid bone marrow sinusoidal capillaries is non-fenestrated, except during the actual process of blood cell transmigration across endothelial cells, after which the transient fenestrations close rapidly behind the transmigrating cells[40-44] (Figure 1, panel D; Additional file 4). Therefore, the transvascular flow of endogenous macromolecules across the capillary wall of myeloid bone marrow sinusoidal blood capillaries into the bone marrow interstitium is via the macula occludens loose junctions of the interendothelial clefts, and the physiologic upper limit of pore size is approximately 5 nm. However, intravenously administered non-endogenous macromolecules larger than this can access the bone marrow interstitium via the phago-endocytic route, which forms the basis of imaging myeloid bone marrow with systemically administered dextran and polyethylene glycol coated nanoparticle-based bone marrow imaging agents[34-38]. These non-endogenous macromolecules, which are approximately 60 nm in diameter, are efficiently phago-endocytosed by the reticuloendothelial cells of the myeloid bone marrow, and upon transvascular release, or spill-over, accumulate within myeloid bone marrow interstitial spaces.

### Sinusoidal non-reticuloendothelial blood capillaries of the spleen: Ultrastructure and the physiologic upper limit of pore size

Two types of sinusoidal blood capillaries exist in the splenic red pulp: the terminal arterial capillaries and venous capillaries (sinuses) (Table 4; not illustrated schematically). Both types are classified as non-reticuloendothelial sinusoidal blood capillaries since the endothelial cells of the endothelial lining of these splenic blood capillaries are of the non-phagocytic phenotype, as they lack a full complement of hydrolytic enzymes, and in this respect, are similar in phenotype to those of the endothelial cell lining of non-sinusoidal blood capillaries[20-22]. The endothelial cell lining of both the terminal arterial capillaries and venous capillaries is coated by a thin endothelial glycocalyx layer[26]. The splenic red pulp arterial capillaries terminate in the red pulp reticular interstitium. The segment of the terminal arterial capillary wall just proximal to the capillary ending in the red pulp is lined by fenestrated endothelial cells[24,27]; however, these endothelial cell fenestrae are covered by the pericapillary macrophage sheath, and therefore, are not functionally open fenestrae[29,30]. The terminal arterial capillary ending openings are approximately 5 μm wide, which permit the passage of plastic microspheres of this size into the red pulp reticular interstitium[28]. Therefore, the physiologic upper limit of pore size of terminal arterial capillary ending openings is approximately 5 μm. The red pulp terminal arterial capillary network constitutes the 'open' slow circulation of the spleen, and is the primary route taken for splenic filtration[28]. The splenic red pulp venous capillaries originate in the splenic red pulp reticular meshwork and drain into the splenic venous system[23,25]. There are very few direct connections between splenic arterial arterioles and splenic venous capillaries; the connections that do exist constitute the 'closed' fast circulation of the spleen, which is the minor route taken for splenic filtration[28]. The interendothelial cell junctions of the tall cuboidal endothelial cells of endothelial lining of splenic red pulp venous capillaries are the apical and basal adherens junctions located 2 to 3 μm apart[23,25]. The interendothelial slits between the apical and basal adherens junctions constitute closed 'potential spaces' across which blood cells migrate, and across which reticulum macrophages extend pseudopodia to phagocytose, for example, nanoparticles within the lumens of venous capillaries[31,32], as belts of the ringed basement membrane layer only exist at apical and basal portions of the lining endothelial cells. It is likely that the closed potential space of the interendothelial slits permits the transvascular convective flow of macromolecules, which have not been phagocytosed at the level of the luminal face of the sinus wall, by the pseudopodia of reticulum macrophages; however, available data is lacking on the subject, the physiologic upper limit of pore size for the interendothelial slits of splenic red pulp venous sinuses has not been defined here.

## Discussion

In the 1940s, the possibility that a thin endothelial glycocalyx layer may exist on the luminal surface of the endothelial cell lining of the blood capillary microvasculature was suggested by Danielli[78], and Chambers and Zweifach[142]; however, at that time, the glycocalyx layer was difficult to visualize by conventional light and electron microscopy staining techniques. Therefore, in 1959, when the morphological classification scheme for vertebrate blood capillaries was developed by Bennett and colleagues[84], the capillary wall was still considered to be a two-layered structure, consisting of the endothelial cell lining and basement membrane layers. Although evidence for the existence of an approximately 20 nm thick glycocalyx layer was provided by Luft in 1966[69] based on electron microscopy of fixed skeletal muscle capillaries stained with ruthenium red, it is now well-known that the endothelial glycocalyx layer is a 150 to 400 nm-thick polysaccharide-rich anionic matrix of sialyated and sulphated proteoglycans and glycoproteins in the physiologic state *in vivo*[61,128]. Since the individual fibers of the glycocalyx are circumferentially spaced a maximum of 20 nm apart[61,128], this relatively narrow interspacing of the individual glycocalyx fibers would restrict the transvascular passage of larger macromolecules through the endothelial glycocalyx layer. This, indeed, appears to be case in the non-sinusoidal fenestrated blood capillaries of the kidney glomeruli that possess open 'non-diaphragmed' fenestrae covered by an intact glycocalyx layer, as the physiologic upper limit of pore size in the capillary wall of this blood capillary type is approximately 15 nm[60], and may be closer to between 13 and 14 nm if the upper limit of pore size of kidney glomerulus capillaries is interrogated using non-flexible spherical macromolecules. This being the case, the barrier to the renal filtration of macromolecules such as hemoglobin (diameter ~6.4 nm) and albumin (diameter ~ 7 nm) are the slit-diaphragms of podocyte foot processes on the abluminal face of the basement membrane layer.

In 1951, Pappenheimer and colleagues formulated the classic small pore theory of microvascular permeability on the basis of experimental data on the restriction to the transvascular flow of various-sized unlabeled endogenous lipid-insoluble molecules across the walls of cat hind-limb microvasculature[6]. By the measurement of the osmotic transients generated by the respective test molecules using the isogravometric osmotic transient technique, Pappenheimer et al. determined that cat hind-limb microvasculature was permeable to inulin (diameter ~3 nm), but not to hemoglobin (diameter ~6.4 nm). On comparison of the experimental values for the restriction to diffusion to theoretically predicted values for uniform water filled cylindrical pores 6 nm in diameter (less restrictive pores), or alternatively, for uniform water-filled rectangular slit-pores approximately 3.7 nm in width (more restrictive pores), it was observed that the experimental data values fit better with the theoretically predicted values for cylindrical pores 6 nm in diameter. However, in lieu of the polydisperse nature of inulin, which was the smaller of the two macromolecular test substances, it was noted that the range of the upper limit of pore size could only be established with additional experimental data demonstrating the restricted transvascular flow of less polydisperse macromolecular test substances similar in size to inulin. In subsequent, similar perfused cat hind-limb osmotic transient experiments with myoglobin (diameter ~4 nm), the restricted transvascular flow of myoglobin was demonstrated, and it was established that the physiologic upper limit of pore size in cat hind-limb blood capillary microvasculature is between 4 and 6 nm[7]. The blood capillary microvasculature of the cat hind-limb is comprised of the capillary microvasculatures of the several different tissue types in the limb, which includes the capillary microvasculatures of skin, muscle, nerve, bone, myeloid bone marrow, adipose, and connective tissues; of these limb tissues, muscle, bone, and adipose tissue capillary microvasculature constitutes one type (non-sinusoidal non-fenestrated) (Table 1; Figure 1, panel A; Additional file 1); skin, nerve, and connective tissue another type (non-sinusoidal fenestrated) (Table 2; Figure 1, panel B; Additional file 2); and myeloid (red) bone marrow yet another (sinusoidal reticuloendothelial) (Table 3; Figure 1, panel D; Additional file 4). The physiologic upper limit of pore size for skin, nerve, and connective tissue blood capillaries, being non-sinusoidal fenestrated capillaries, is within the 6 to 12 nm range (Table 2). Therefore, the physiologic upper limit of pore size of cat hind-limb blood capillary microvasculature, as measured by the isogravometric osmotic transient method, would be a slight over-estimation of the 'actual' physiologic upper limit of pore size in skeletal muscle blood capillary microvasculature. This is the likely reason why the experimental data values fit better with the theoretically predicted values for aqueous cylindrical pores 6 nm in diameter. This conclusion is supported by the ultrastructural evidence that the interendothelial cell clefts in the capillary walls of the non-sinusoidal non-fenestrated skeletal muscle blood capillaries are lined by macula occludens interendothelial cell junctions, which, in series, restrict the transcapillary passage of macromolecules larger than horseradish peroxidase (diameter ~4.6 nm) across the interendothelial cell cleft[79](Table 1; Figure 1, panel A; Additional file 1).

The interplay of tissue blood capillary and interstitial space hydrostatic and oncotic pressures favors the net filtration of fluids and lipid-insoluble molecules across the blood capillary walls, and this ultrafiltrate, is the tissue lymph[47,143-147]. Since the openings of the terminal endings of tissue interstitium lymphatic capillaries, or initial lymphatics, are permeable to macromolecules upwards of several hundred nanometers, the level of the restriction to the passage of systemically administered macromolecules is the tissue blood capillary wall[148-151]. These differences in the ultrastructure of the tissue blood capillary and lymphatic capillary walls is the basis on which differences in macromolecule plasma concentration and regional lymph concentration have been used as indexes of regional differences in blood capillary permeability. Non-endogenous macromolecules that are not rapidly cleared from blood circulation accumulate in the myeloid bone marrow interstitium and the periosteal fibrous tissue lymphatics[47-49]. This information is most pertinent to the interpretation of lower extremity and cervical region lymph flow data as the presence of non-endogenous macromolecules larger than approximately 5 nm in diameter within the lymphatic drainage of these regions would be attributable to the accumulation of these non-endogenous macromolecules in the myeloid bone marrow sinusoidal transcapillary filtrate.

Upon the intravenous administration of radiolabeled native macromolecules, radio-iodinated albumin and immunoglobulin, to dogs with cannulated thoracic lymph ducts, and the measurement of the changes in the respective test molecule concentrations in plasma and thoracic duct lymph, Wasserman and Mayerson, in 1952, noted that: (1) the lymph concentration of radio-iodinated albumin increases approximately 1.6 times faster than that of radio-iodinated immunoglobulin; and that (2) when administered at high-doses, steady-state lymph levels of both radio-iodinated species are achieved within 90 minutes after administration; whereas, when administered at low-doses, steady-state lymph levels are achieved between 7 and 13 hours after administration[11]. The faster rate of accumulation of radio-iodinated albumin than immunoglobulin in thoracic duct lymph is consistent with the fact that the pore sizes in the diaphragmed fenestrae of non-sinusoidal fenestrated blood capillaries in many visceral organs and tissues are more restrictive to the transcapillary passage of immunoglobulin than albumin, as the physiologic upper limits of pore size in non-sinusoidal fenestrated blood capillaries with diaphragmed fenestrae vary between 6 and 12 nm (Table 2). However, the fact that the test substances employed were radiolabeled native macromolecules is notable, as these radio-iodinated test substances[54] constitute non-endogenous macromolecules that would be phago-endocytosed by reticuloendothelial cells of hepatic and myeloid bone marrow sinusoidal capillary walls. Based on the observed dose-related differences in the rates of radio-iodinated albumin and immunoglobulin accumulation in the thoracic duct lymphatic drainage, which is primarily that of the hepatic region lymph, the great proportion of these non-endogenous macromolecules the administered at low-doses are likely phago-endocytosed at the level of the hepatic sinusoidal blood capillary walls, and do not have the opportunity to actually flow across the open fenestrae of the hepatic sinusoids to enter the hepatic interstitium by the transvascular convective route. However, at high doses, the phago-endocytic activity threshold of the lining reticuloendothelial cells of the hepatic sinusoid blood capillaries is reached; and as such, the proportion of the dose of the circulating macromolecules above and beyond the phago-endocytic activity threshold flows across the open fenestrae and enters the hepatic interstitial space via the transvascular convective route. Therefore, at high doses, the radio-iodinated albumin and immunoglobulin accumulate in the hepatic region lymph and in thoracic lymphatic drainage at faster rates, than when administered at low-doses.

In 1956, Grotte et al. performed a series of additional dog lymph flow studies by employing various-sized dextran and plastic nanoparticles, the findings of which were the basis for the formulation of the dual pore hypothesis of microvascular permeability[12,13]. The experimental findings underlying the formulation of the dual pore hypothesis of capillary permeability are discussed herein from the physiologic perspective. When the findings are viewed in this light, it becomes apparent that the findings are not *per se *a confirmation for the existence of a large pore population, but rather, are evidence for the role of the reticuloendothelial cells of the hepatic and myeloid bone marrow sinusoids in the phago-endocytosis non-endogenous macromolecules. Grotte employed dextran nanoparticles ranging in size from approximately 2.5 to 24 nm in diameter, and fluorescent spherical plastic (methylmethacrylate) nanoparticles ranging in size from 60 to 140 nm in diameter, and measured the steady-state concentrations of the respective non-endogenous macromolecules in the locoregional lymphatics; which, in case of the lymph flow studies with the dextrans were the hepatic, lower extremity, and cervical region lymphatics; and in the case of the plastic nanoparticles were the hepatic, lower extremity, cardiac, and bronchial region lymphatics. The lymph concentrations of the dextrans smaller than 8 to 10 nm in diameter were measured at 7 hours after the intravenous infusion to animals with renal occlusion, and those of the larger dextrans were measured at 24 hours, which would have been sufficient periods of time for the smaller and larger dextrans to have reached steady-state lymph concentrations. It is notable that the lymph concentrations of the plastic nanoparticles were measured immediately following infusions at various time points over a period of between 3 and 4 hours, since these larger plastic nanoparticles were quickly cleared from systemic blood circulation, which is attributable to the rapid removal of these immunogenic particles by phago-endocytic uptake by hepatic Kupffer macrophages, and splenic red pulp macrophages, as well as, the reticuloendothelial cells of the hepatic sinusoidal capillaries.

On review of lymph flow study experimental data, it is evident that the point at which the particle plasma:lymph concentration ratio first deviates from approximately 1, or unity, represents the pore size cut-off of the capillary ultrafiltrate and the lymphatic drainage of the capillary population with the lowest upper limit of pore size, which constitutes that of non-sinusoidal non-fenestrated capillaries; and, several graded decreases in the particle plasma:lymph concentration ratio over a range of particle sizes represent the pore size cut-offs of the capillary ultrafiltrates of multiple capillary populations with different upper limits of pore size, which constitutes that of fenestrated capillaries. In case of the lower extremity and cervical region lymphatic drainage, there are graded decreases in the dextran particle plasma:lymph concentration ratios between dextran particle sizes from 4 to 8 nm in diameter, from approximately 1 to approximately 0.15 for lower extremity lymph, and from approximately 1 to approximately 0.25 for cervical lymph, which indicates the presence of populations of non-sinusoidal non-fenestrated capillaries with the upper limit of pore size closer to 4 nm, and indicates the presence of populations of non-sinusoidal fenestrated capillaries in the region with the upper limit of pore size closer to 8 nm. In the case of the hepatic region lymphatic drainage, the dextran particle plasma:lymph concentration ratio decreases from approximately 1 to approximately 0.85 at a dextran particle size of 8 nm, which indicates the presence of a population of non-sinusoidal fenestrated capillaries with a physiologic upper limit of pore size of approximately 8 nm, consistent with the cut-off of pore size of the capillary ultrafiltrate of intestinal mucosal non-sinusoidal fenestrated blood capillaries (Table 2).

The observation that the plasma:lymph concentration ratio for the larger dextran particles between 8 and 24 nm in diameter remains unchanged for various body regions has been cited as physiologic evidence for the existence of another population of large pores, or 'capillary leaks', at least 24 nm in diameter in blood capillary microvasculature. Further, this observation, coupled with the finding that the plastic particle plasma:lymph concentration ratio for the lower extremity, cardiac, and bronchial region lymphs is approximately 0, or unmeasurable, has been cited as the physiologic evidence for the existence of the large pore population in capillary microvasculature. Based on these findings, that particles as large as 24 nm accumulate in the lower extremity and cervical lymph (dextran nanoparticles), and that particles 60 nm, and larger, do not accumulate in the lower extremity and cervical lymph (plastic nanoparticles), it has been concluded that the physiologic upper limit of pore size of the large pore population in capillary microvasculature ranges between 24 and 60 nm. Furthermore, the differences in the regional dextran particle plasma:lymph concentration ratios for the 8 to 24 nm diameter dextran particles (approximately 0.15 for lower extremity region lymph, approximately 0.25 for cervical region lymph, and approximately 0.85 for hepatic region lymph) have been additionally cited as evidence for differences in the number of small and large pores in the blood capillary populations of the lower extremity, cervical, and hepatic regions, with the small-to-large pore ratios for the respective regions being 1:34,000, 1:18,000, and 1:340[12].

In case of the dextran nanoparticles between 8 and 24 nm in diameter, the findings taken altogether, support the likelihood that the administered dose of the dextran nanoparticles was high enough to saturate the phago-endocytic capacity of the reticuloendothelial cells of the hepatic and myeloid bone marrow sinusoidal capillaries, resulting in the accumulation of the dextrans nanoparticles in the respective interstitial tissue spaces and regional lymph. With regards to the accumulation in the hepatic interstitium and lymph, this would be via transvascular flow through open fenestrae and the phago-endocytic route; and with regards to the myeloid bone marrow, this would be via the phago-endocytic route. It is postulated here that the measured dextran nanoparticle concentrations in the lower extremity and cervical regional lymphatic drainages constitute the dextran nanoparticle concentrations of the myeloid bone marrow sinusoidal capillary filtrates of the respective regions. The relatively high dextran particle plasma:lymph concentration ratio of the hepatic region lymph (approximately 0.85), as compared to that of lower extremity and cervical region lymphs (approximately 0.2), is consistent with the hepatic sinusoidal capillary filtrate constituting the major proportion of the lymphatic drainage of the hepatic region. In case of the plastic nanoparticles between 60 and 140 nm, the particle plasma:lymph concentration ratio for the hepatic region lymph only reaches approximately 0.2 over 3 hours, and does not maintain steady-state levels over longer time periods; whereas particle plasma:lymph concentration ratio for the lower extremity, cardiac, and bronchial region lymph is approximately 0, or unmeasurable. The relatively rapid clearance of the plastic particles from blood circulation is attributable to the rapid sequestration of these particles in the splenic red pulp followed by phagocytosis of particles by the pulp macrophages. Therefore, the low level of plastic particle accumulation in the hepatic interstitium and associated lymphatic drainage, and virtually no plastic particle accumulation in the lower extremity, cardiac, and bronchial region lymphatic drainages, reflects the low level of phago-endocytic particle uptake at the level of the myeloid bone marrow blood capillaries, secondary to the rapid clearance of plastic particles from systemic blood circulation.

## Conclusions

Tissue blood capillaries have been classified here on the basis of the capillary type-specific differences in the physiologic upper limit of pore size to the transcapillary passage of lipid-insoluble molecules. When tissue blood capillaries are classified on the basis of capillary wall ultrastructural differences, as these differences relate to function, it becomes evident that the primary route for the transcapillary exchange of lipid-insoluble small molecules and macromolecules across the capillary walls of blood capillaries, other than those of myeloid bone marrow blood capillaries, is via transvascular flow of the molecules through aqueous small pores, and that the upper limit of pore size in the capillary walls of these blood capillaries is the physiologic upper limit of pore size for transcapillary transport of lipid-insoluble molecules. In case of myeloid bone marrow blood capillaries, the upper size limit to transvascular transport of lipid-insoluble molecules across the capillary wall is dependent on the route of the transvascular transport, since the transcapillary passage of lipid-insoluble macromolecules smaller than 5 nm in diameter can take place through aqueous small pores in the interendothelial cell junctions; whereas, that of larger non-endogenous macromolecules takes place upon phago-endocytosis of the macromolecules by reticuloendothelial cells and then the release of endocytosed molecules across the capillary wall into the myeloid bone marrow interstitium.

The physiologic upper limit of pore size in the capillary walls of most non-sinusoidal blood capillaries to the transcapillary passage of lipid-insoluble endogenous and non-endogenous macromolecules ranges between 5 and 12 nm. Therefore, macromolecules larger than the physiologic upper limits of pore size in the non-sinusoidal blood capillary types generally do not accumulate within the respective tissue interstitial spaces and their lymphatic drainages. In the case of reticuloendothelial sinusoidal blood capillaries of myeloid bone marrow, however, non-endogenous macromolecules as large as 60 nm in diameter can distribute into the bone marrow interstitial space via the phago-endocytic route, and then subsequently accumulate in the locoregional lymphatic drainages of tissues following absorption into the lymphatic drainage of periosteal fibrous tissues, which is the lymphatic drainage of myeloid bone marrow. When the ultrastructural basis for transcapillary exchange across the capillary walls of different capillary types is viewed in this light, it becomes evident that the physiologic evidence for the existence of aqueous large pores ranging between 24 and 60 nm in diameter in the capillary walls of blood capillaries, is circumstantial, at best.

## Competing interests

The authors declare that they have no competing interests.

## Authors' contributions

HS conceptualized the work and wrote the manuscript.

## Supplementary Material

Additional file 1 **Figure **1** panel A with detailed description**
For non-sinusoidal non-fenestrated blood capillaries, the pore size of the interendothelial cell junction openings delineates the physiologic upper limit of pore size in the capillary wall, which is < 1 nm for non-sinusoidal non-fenestrated tissue blood capillaries with zona occludens junctions (i.e. brain and spinal cord), and approximately 5 nm for non-sinusoidal non-fenestrated tissue blood capillaries with macula occludens junctions (i.e. skeletal muscle). Click here for file

Additional file 2 **Figure **1** panel B with detailed description**
For non-sinusoidal fenestrated blood capillaries with diaphragmed fenestrae, the pore size of the open spaces within the fenestrae devoid of membranous components (central diaphragm [shown in red] and the septae of the diaphragm that radiate outward to the fenestral rim [not shown]) delineates the physiologic upper limit of pore size, which ranges between 6 and 12 nm. Click here for file

Additional file 3 **Figure **1** panel C with detailed description**
In the case of non-sinusoidal fenestrated blood capillaries with open 'non-diaphragmed' fenestrae, the only known healthy tissue with this blood capillary type is the kidney glomerulus. The pore size of the open spaces between the individual glycocalyx matrix fibers in the vicinity of the fenestrae (shown in red) delineates the physiologic upper limit of pore size, which is approximately 15 nm. Click here for file

Additional file 4 **Figure **1 **panel D with detailed description**
In the case of sinusoidal reticuloendothelial non-fenestrated blood capillaries of myeloid bone marrow, the lining reticuloendothelial cells of myeloid bone marrow sinusoidal blood capillaries are only fenestrated during the actual process of blood cell transmigration, as is depicted in panel D. Since these 'cellular transmigration pores' close immediately following cellular transit, and the endothelial cells are not permanently fenestrated, the endothelial cells are non-fenestrated with respect to the transvascular flow of macromolecules. The pore size of the openings in the macula occludens interendothelial cell junctions is the primary determinant of the physiologic upper limit of pore size to the transvascular flow of macromolecules, which is ~5 nm. Non-endogenous macromolecules larger than 5 nm in diameter with long blood half-lives, which are not rapidly phagocytosed by macrophages (hepatic Kupffer and splenic red pup macrophages), accumulate in the bone marrow interstitium upon the transvascular release of phago-endocytosed particles into the marrow interstitium. Click here for file

Additional file 5 **Figure **1 **panel E with detailed description**
In the case of sinusoidal reticuloendothelial fenestrated blood capillaries of the liver, the capillary wall of hepatic sinusoidal blood capillaries is lined by reticuloendothelial cells with open fenestrae of relatively wide diameters, which can be on the order of 180 nm (humans) to 280 nm (rodents). Due to the lack of an appreciable concentration of glycocalyx matrix fibers in the vicinity of the fenestral openings, and an absence of the basement membrane layer, the physiologic upper limit of pore size in the hepatic sinusoidal capillary wall is approximately the pore size of the fenestral openings, which permit the unrestricted transvascular flow of smaller chylomicrons and lipoproteins into the hepatic interstitium. Non-endogenous macromolecules with long blood half-lives can access the hepatic interstitium either via transvascular flow across the open fenestrae or upon the transvascular release of macromolecules phago-endocytosed by capillary wall reticuloendothelial cells and hepatic Kupffer macrophages. Click here for file
