# Supplementary figures and images for: Physiologic upper limits of pore size of different blood capillary types and another perspective on the dual pore theory of microvascular permeability
Source: J Angiogenes Res. 2010 Aug 11;2:14. doi: 10.1186/2040-2384-2-14 (PMC2928191; doi:10.1186/2040-2384-2-14)

# A. Non-Sinusoidal Non-Fenestrated

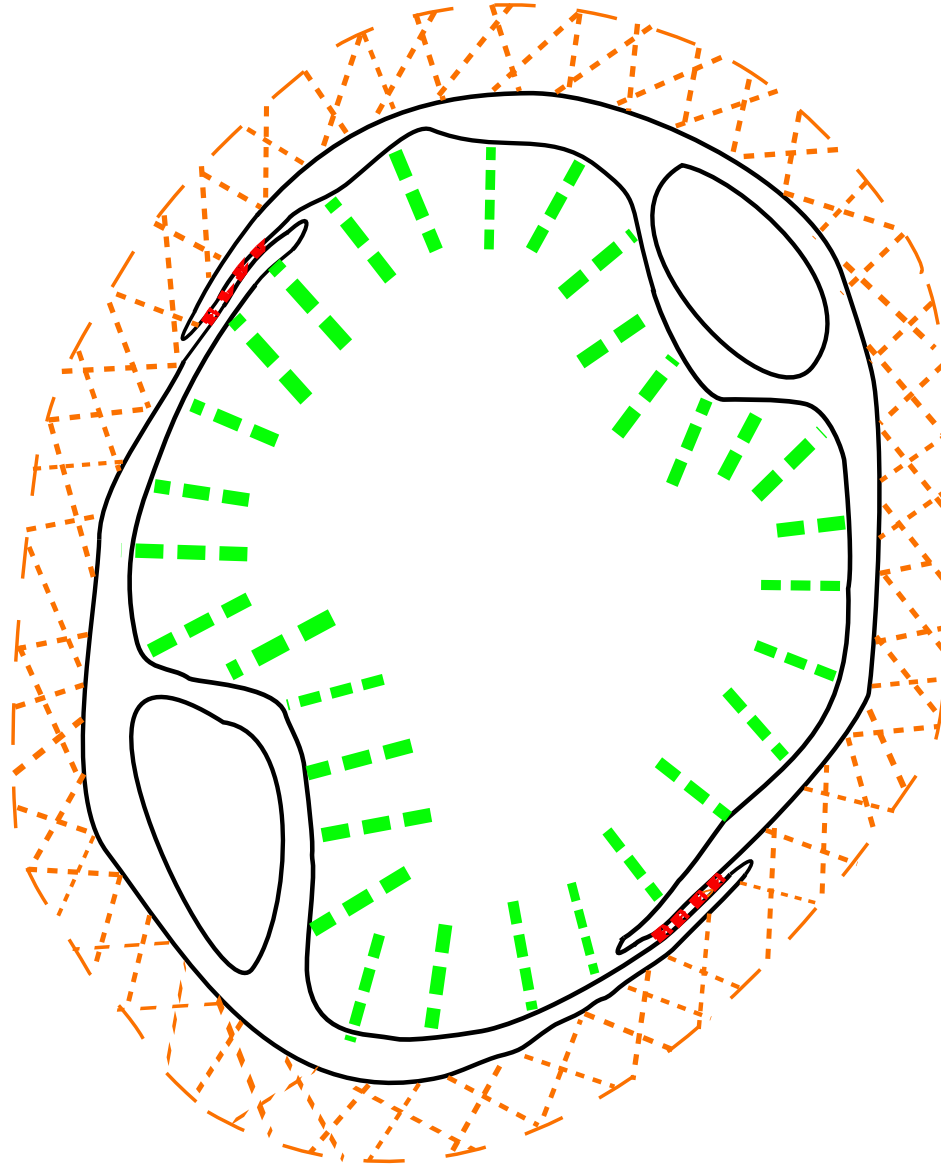

Supplement: Additional file 1 — Figure 1 panel A with detailed description For non-sinusoidal non-fenestrated blood capillaries, the pore size of the interendothelial cell junction openings delineates the physiologic upper limit of pore size in the capillary wall, which is < 1 nm for non-sinusoidal non-fenestrated tissue blood capillaries with zona occludens junctions (i.e. brain and spinal cord), and approximately 5 nm for non-sinusoidal non-fenestrated tissue blood capillaries with macula occludens junctions (i.e. skeletal muscle). [file 2040-2384-2-14-S1.PDF]

**B.** Non-Sinusoidal Fenestrated  
(Diaphragmed Fenestrae)

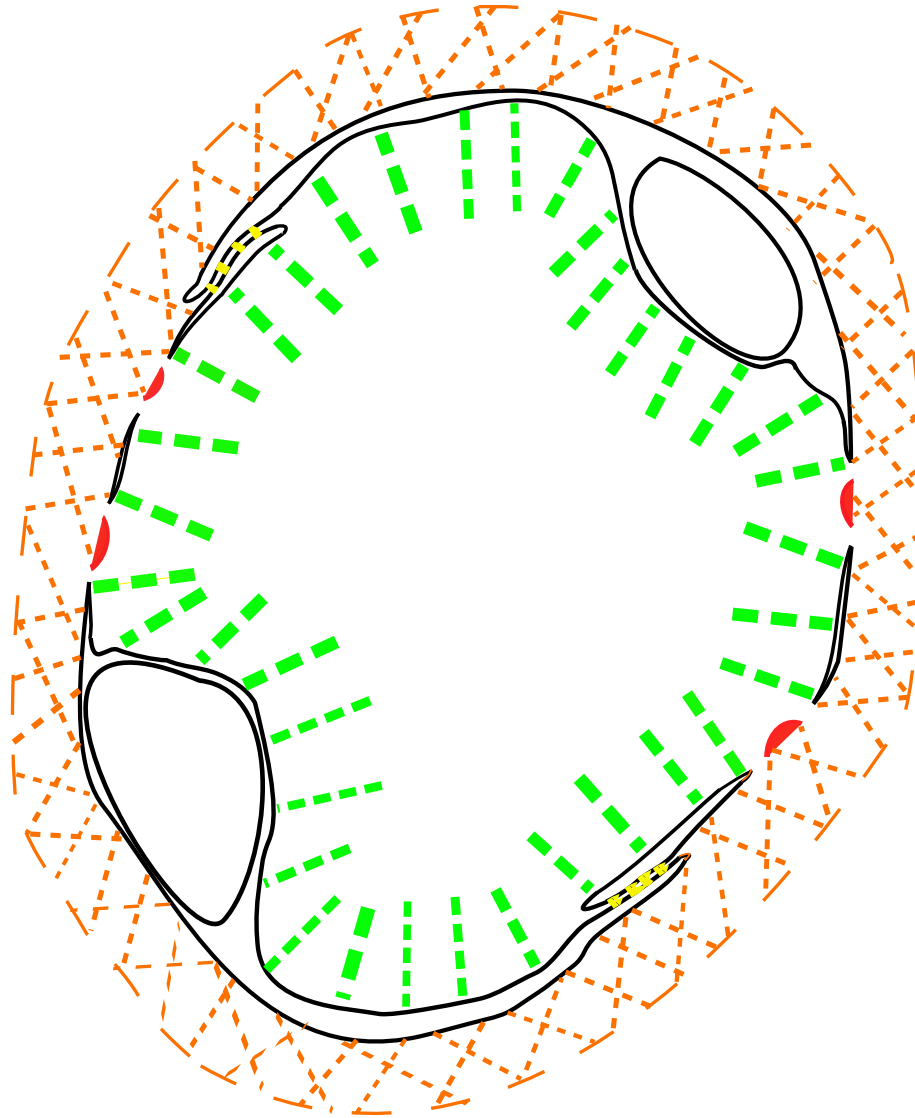

Supplement: Additional file 2 — Figure 1 panel B with detailed description For non-sinusoidal fenestrated blood capillaries with diaphragmed fenestrae, the pore size of the open spaces within the fenestrae devoid of membranous components (central diaphragm [shown in red] and the septae of the diaphragm that radiate outward to the fenestral rim [not shown]) delineates the physiologic upper limit of pore size, which ranges between 6 and 12 nm. [file 2040-2384-2-14-S2.PDF]

# C. Non-Sinusoidal Fenestrated (Open Fenestrae)

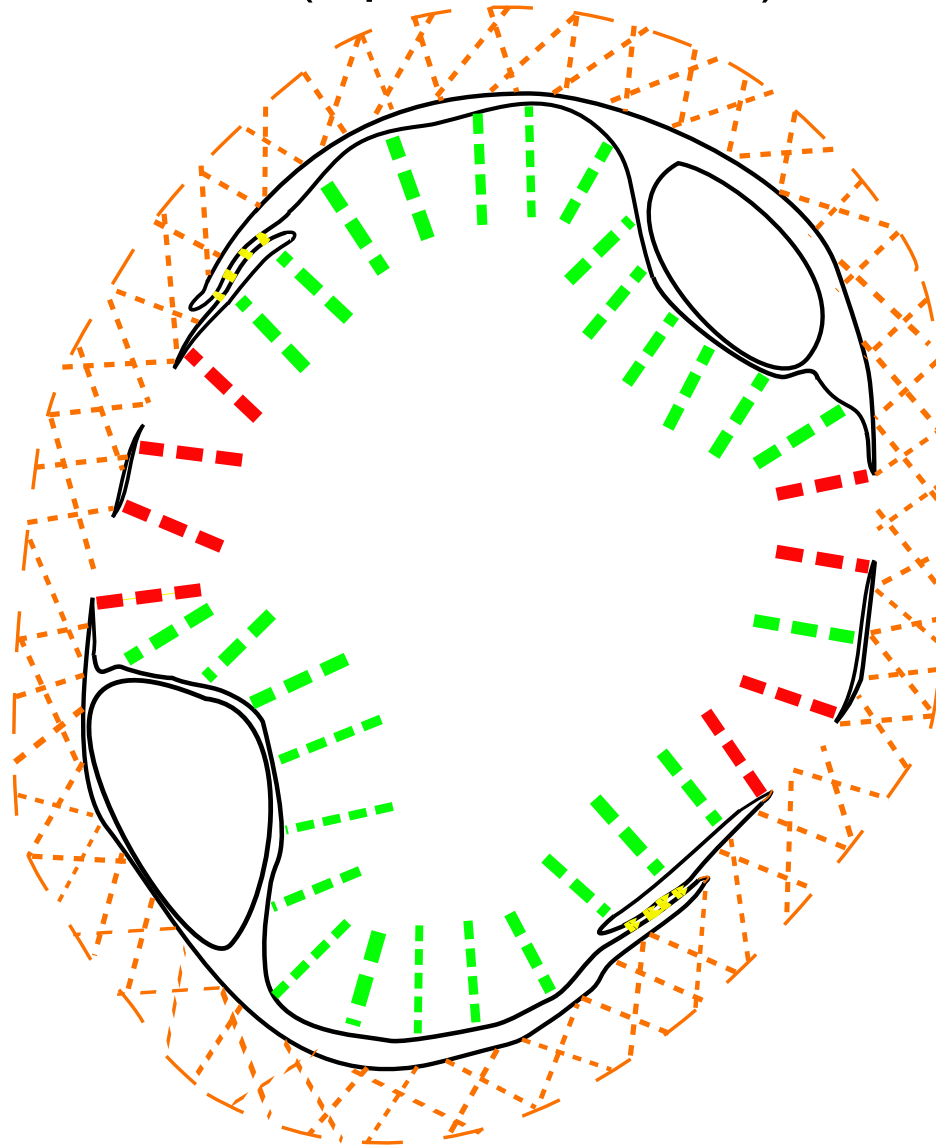

Supplement: Additional file 3 — Figure 1 panel C with detailed description In the case of non-sinusoidal fenestrated blood capillaries with open 'non-diaphragmed' fenestrae, the only known healthy tissue with this blood capillary type is the kidney glomerulus. The pore size of the open spaces between the individual glycocalyx matrix fibers in the vicinity of the fenestrae (shown in red) delineates the physiologic upper limit of pore size, which is approximately 15 nm. [file 2040-2384-2-14-S3.PDF]

**D** Sinusoidal Reticuloendothelial Non-Fenestrated  
▪ (Myeloid Bone Marrow)

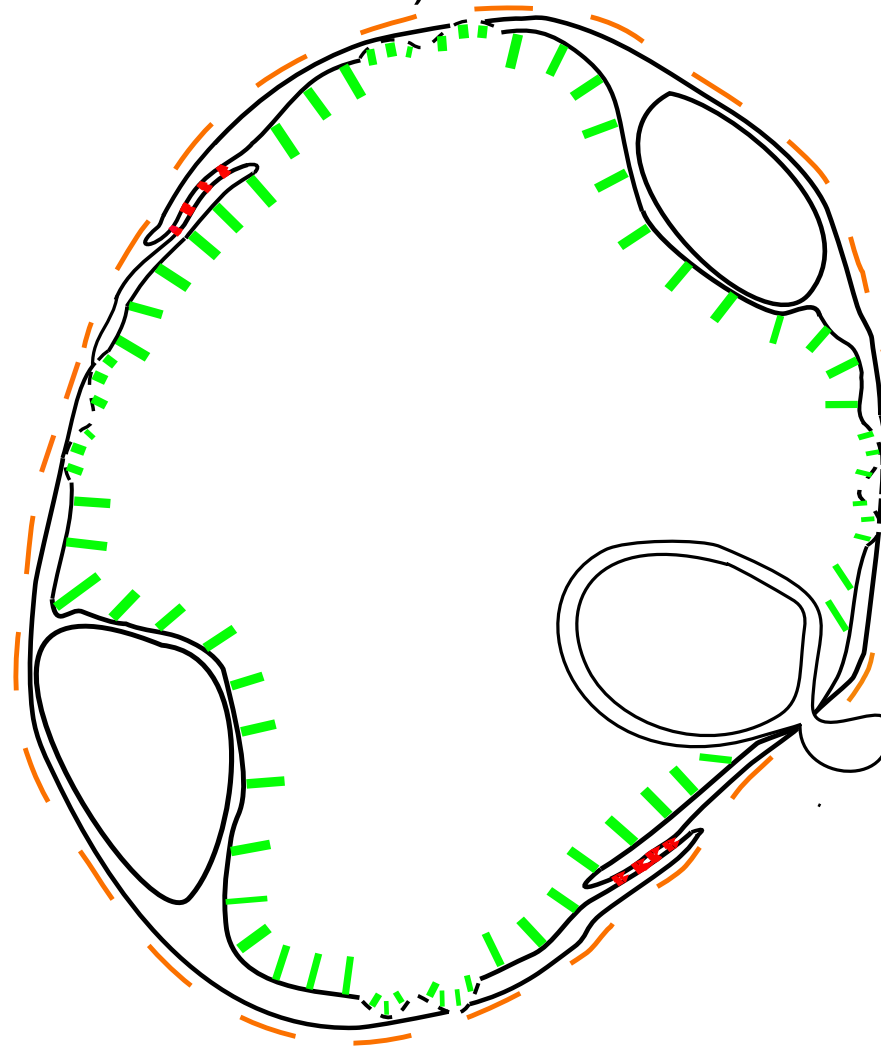

Supplement: Additional file 4 — Figure 1 panel D with detailed description In the case of sinusoidal reticuloendothelial non-fenestrated blood capillaries of myeloid bone marrow, the lining reticuloendothelial cells of myeloid bone marrow sinusoidal blood capillaries are only fenestrated during the actual process of blood cell transmigration, as is depicted in panel D. Since these 'cellular transmigration pores' close immediately following cellular transit, and the endothelial cells are not permanently fenestrated, the endothelial cells are non-fenestrated with respect to the transvascular flow of macromolecules. The pore size of the openings in the macula occludens interendothelial cell junctions is the primary determinant of the physiologic upper limit of pore size to the transvascular flow of macromolecules, which is ~5 nm. Non-endogenous macromolecules larger than 5 nm in diameter with long blood half-lives, which are not rapidly phagocytosed by macrophages (hepatic Kupffer and splenic red pup macrophages), accumulate in the bone marrow interstitium upon the transvascular release of phago-endocytosed particles into the marrow interstitium. [file 2040-2384-2-14-S4.PDF]

# E. Sinusoidal Reticuloendothelial Fenestrated (Hepatic)

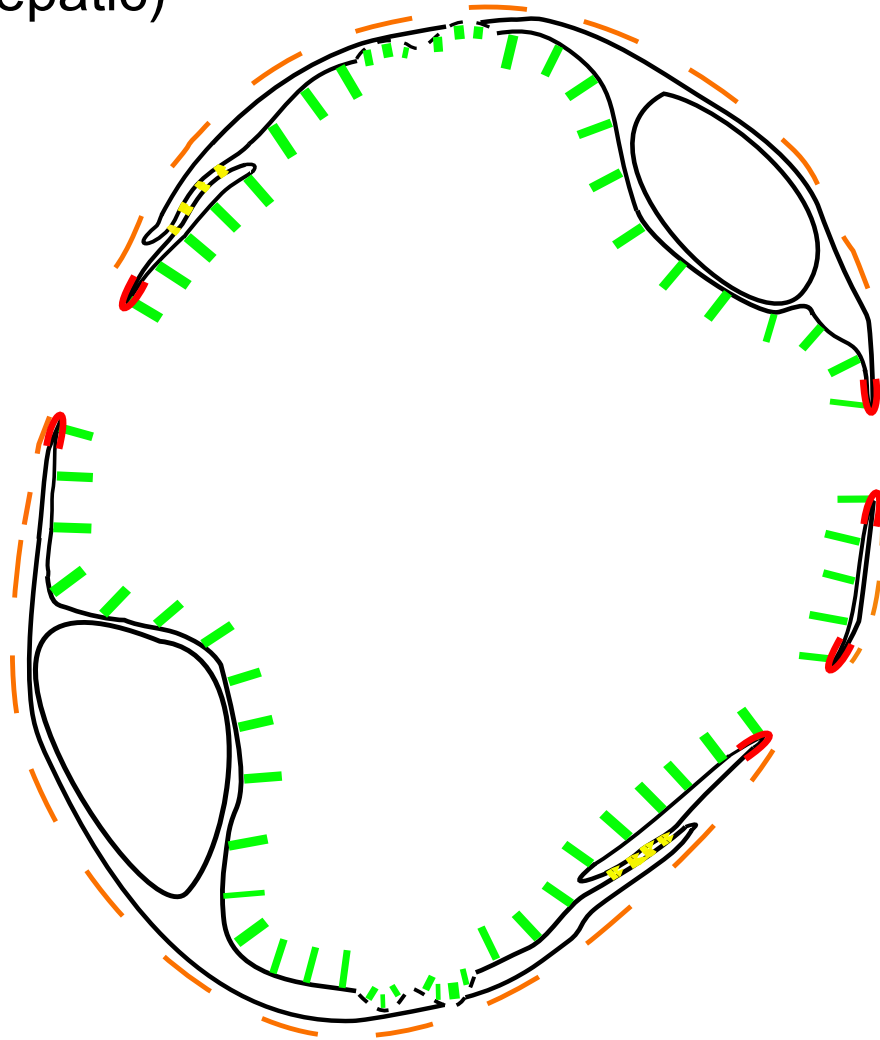

Supplement: Additional file 5 — Figure 1 panel E with detailed description In the case of sinusoidal reticuloendothelial fenestrated blood capillaries of the liver, the capillary wall of hepatic sinusoidal blood capillaries is lined by reticuloendothelial cells with open fenestrae of relatively wide diameters, which can be on the order of 180 nm (humans) to 280 nm (rodents). Due to the lack of an appreciable concentration of glycocalyx matrix fibers in the vicinity of the fenestral openings, and an absence of the basement membrane layer, the physiologic upper limit of pore size in the hepatic sinusoidal capillary wall is approximately the pore size of the fenestral openings, which permit the unrestricted transvascular flow of smaller chylomicrons and lipoproteins into the hepatic interstitium. Non-endogenous macromolecules with long blood half-lives can access the hepatic interstitium either via transvascular flow across the open fenestrae or upon the transvascular release of macromolecules phago-endocytosed by capillary wall reticuloendothelial cells and hepatic Kupffer macrophages. [file 2040-2384-2-14-S5.PDF]
